# Impact of high hydrostatic pressure on the cytokine profile and head and neck cancer cell behavior: implications for oncological safety

**DOI:** 10.3389/fimmu.2025.1581014

**Published:** 2025-07-28

**Authors:** Julia Kristin Brach, Vivica Freiin Grote, Daniel Strüder, Friederike Kalle, Anika Jonitz-Heincke, Rainer Bader, Marco Hoffmann, Agmal Scherzad, Markus Wirth, Stephan Hackenberg

**Affiliations:** ^1^ Department of Otorhinolaryngology-Head and Neck Surgery, University Hospital RWTH Aachen, Aachen, Germany; ^2^ Center for Integrated Oncology (CIO), University Hospital RWTH Aachen, Aachen, Germany; ^3^ Research Laboratory for Biomechanics and Implant Technology, Department of Orthopedics, Rostock University Medical Centre, Rostock, Germany; ^4^ Department of Otorhinolaryngology, Head and Neck Surgery “Otto Körner”, Rostock University Medical Center, Rostock, Germany; ^5^ Department of Urology and Pediatric Urology, University Hospital RWTH Aachen, Aachen, Germany; ^6^ Department of Oto-Rhino-Laryngology, Plastic, Aesthetic and Reconstructive Head and Neck Surgery, University of Wuerzburg, Wuerzburg, Germany

**Keywords:** high hydrostatic pressure (HHP), cytokine release, head and neck cancer, whole cell vaccination, immune modulation, squamous cell carcinoma

## Abstract

**Introduction:**

The devitalization of tissue using high hydrostatic pressure (HHP) is an advanced method for processing tumor-infiltrated cartilage to treat tissue defects. This approach preserves the structural and biomechanical properties of the graft while effectively eliminating tumor cells. However, HHP induces the release of cytokines, which may influence the behavior of residual tumor cells in the surrounding tissue. This study characterizes cytokine profiles of HHP-treated head and neck squamous cell carcinoma (HNSCC) cell lines and evaluated its biological effects on intact tumor cells to further assess the oncological safety of the method.

**Methods and results:**

HHP- treatment resulted in a dose-dependent release of pro-inflammatory cytokines, primarily IL-1α and IL-1β, in all investigated cell lines, while IL-6 and IL-8 concentrations were higher in untreated samples. Functional assays demonstrated that supernatants from HHP-treated HNSCC cells significantly enhanced proliferation, migration, and invasion of HNSCC cells relative to control conditions, with these effects being most pronounced at 200 MPa, a pressure associated with incomplete tumor cell devitalization. At 300 MPa, HHP achieved complete devitalization, correlating with intensified necrotic processes and increased intracellular cytokine release.

**Discussion:**

Our findings indicate that while HHP significantly influences the cytokine profile and tumor cell behavior, pressures of ≥300 MPa ensure complete tumor cell devitalization, supporting its oncological safety for clinical applications. Further *in vivo* studies are needed to validate these observations and confirm the clinical safety of HHP-treated materials.

## 1 Introduction

High Hydrostatic Pressure (HHP) is an emerging technique in tissue engineering and regenerative medicine, offering a cost-effective approach for tissue devitalization while preserving structural integrity and biomechanical properties (1–6). By applying pressures up to 600 MPa, HHP disrupts cellular membranes, leading to cell devitalization without compromising the extracellular matrix, a critical factor for the success of grafts and implants in reconstructive surgery (1, 7). Due to its uniform pressure distribution across complex tissues according to Pascal´s law, HHP ensures standardized treatment, making it especially valuable for the preparation of autologous, tumor-infiltrated tissues such as cartilage for reimplantation (1, 3, 8).

While HHP is extensively used in food safety application for bacterial inactivation, its potential in regenerative medicine and oncology remains an active area of research (1, 9–12). Initial approaches from our working group have explored the use of advanced tissue grafts in head and neck surgery, where reconstruction is performed utilizing HHP-treatment (13). In head and neck surgery, especially for squamous cell carcinomas (SCC), functional and aesthetic preservation is a crucial aspect (14–18). SCCs in the head and neck region often cause significant tissue damage, have high recurrence rates, and often respond inadequately to secondary treatments such as immunotherapy (19–24). This challenge is particularly evident in cases of laryngeal infiltration, where partial laryngeal resection may be necessary for organ preservation. However, effective reconstruction is often limited by the loss of structural support within the laryngeal skeleton. Autologous reconstruction of tumor-surrounding or even partially affected cartilage might provide a tissue-preserving option that addresses both functional and aesthetic concerns (25–27).

Beyond tissue devitalization, HHP-treatment of HNSCC cells has demonstrated its capacity to induce immunogenic cell death, marked by calreticulin translocation and elevated ATP release at higher pressures, which can stimulate an antigen-specific immune response (28). This positions HHP as a valuable tool in developing whole-cell cancer vaccines. HHP provides an efficient and non-toxic method for devitalizing tumor cells, serving as an alternative to conventional methods such as chemical treatment, irradiation, or freeze-thaw cycles (29, 30). Importantly, HHP-treatment preserves the antigenicity of tumor cells, as well as the primary and secondary structures of proteins (28, 30, 31). Studies have shown that dendritic cell-based vaccines, pulsed with HHP-inactivated tumor cells, hold promise as an immunotherapy approach for solid tumors (30, 32–35). Additionally, it was observed that HHP-treated melanoma cells, in combination with irradiation, could significantly suppress tumor growth in mice (8, 36–38).

A previous work has shown that HHP-treatment at 315 MPa for 10 min effectively devitalizes head and neck SCC (HNSCC) cells while preserving the integrity of the cartilage matrix (28). While this safe devitalization of tumor cells is promising, ensuring the oncological safety of autografts for reimplantation remains crucial. In particular, residual cytokines and tumor antigens within the treated cartilage could influence surrounding cells, including any remaining tumor cells, stem cells, and fibroblasts. Cytokines play a central role as mediators of the immune response and may be critical in the context of HHP-treatment. Pro-inflammatory cytokines such as Interleukin (IL)-1, IL-6, and IL-8 are of particular interest, as they can influence the tumor microenvironment and potentially stimulate immune responses (39–41). However, they may also contribute to chronic inflammation, a factor associated with tumor progression in SCCs (39). Conversely, certain cytokines can act as adjuvants in tumor vaccination by enhancing antigen presentation, promoting T-cell responses, or modulating the immune environment in ways that support anti-tumor immunity (42–45).

A detailed analysis of the cytokines released during HHP-treatment is crucial to evaluate how these factors impact the tumor microenvironment and oncological safety. In this study, relevant cytokines were identified through comprehensive screening, and their release during HHP-treatment was analyzed. Experiments were conducted to assess the effects of these cytokines on the proliferation, migration, and invasion of HNSCC cell lines. These investigations are essential to fully understand the potential of HHP-treatment for autologous reconstruction and the development of immunotherapies.

## 2 Materials and methods

**Figure 1:**
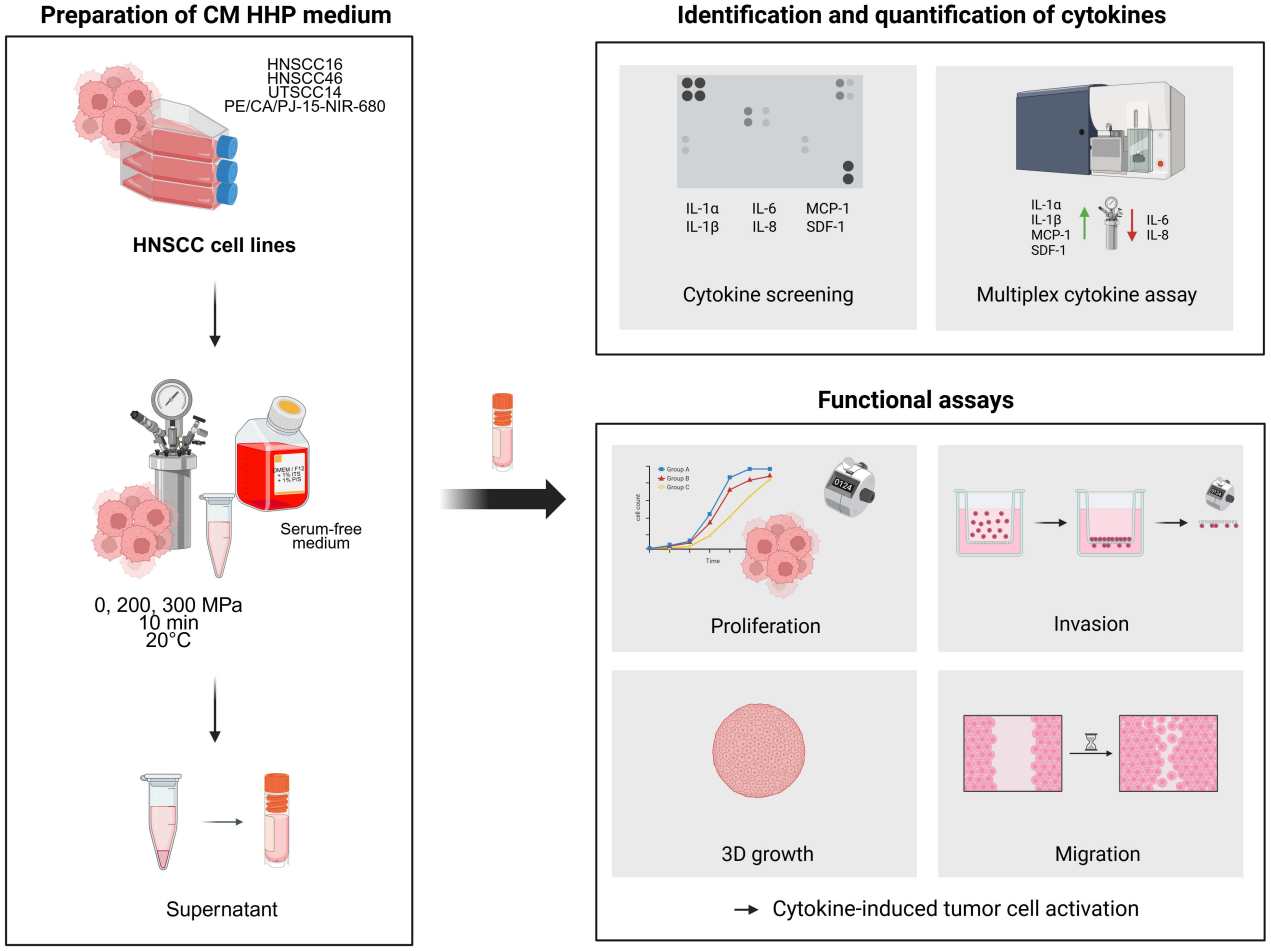
Experimental workflow and key findings of the study. HNSCC cell lines (HNSCC16, UTSCC14, HNSCC46 and PE/CA/PJ-15-NIR-680) were treated with high hydrostatic pressure (0, 200, 300 MPa), and the resulting conditioned media (CM) were analyzed for cytokine release using dot blot and multiplex assays. Identified cytokines (IL-1α, IL-1β, IL-6, IL-8, MCP-1, SDF-1) were functionally assessed for their effects on tumor cell proliferation, migration, and invasion.

The experimental procedures used to investigate cytokine release following HHP treatment of HNSCC cells and the subsequent effects of conditioned media (CM) on tumor cell behavior are summarized in the graphical abstract shown in **Figure 1**.

### 2.1 HNSCC cell line cultivation

The head and neck squamous carcinoma cell lines HNSCC16 (larynx P1 M1), UTSCC14 (tongue, RRID: CVCL_7810), HNSCC46 (hypopharynx P0 M2) and PE/CA/PJ-15 (tongue, RRID: CVCL_2678) were utilized in this study.

PE/CA/PJ-15, originally obtained from the German collection of cell cultures (DSMZ; Braunschweig, Germany) was transduced to stably express the fluorescent near-infrared protein iRFP680 (NIR). The primary tumor cells HNSCC16 and HNSCC46 were established by the research group (Schoenwalder et al. (46)) and authenticated by STR profiling (against the respective patient tumor and patient-derived xenograft (PDX)). All cell lines were Human papillomavirus (HPV) negative, and all experiments were performed with mycoplasma-free cells.

Cells were grown in Dulbecco´s modified Eagel medium (DMEM)/Ham´s F12 (1:1) (Pan Biotech Germany) supplemented with 10% fetal calf serum (FCS, Pan Biotech, Germany) and 1% Penicillin/Streptomycin (P/S, Sigma-Aldrich, US) and cultured in 75 cm^2^ culture flasks at 37°C with 5% CO_2_ in a humified incubator. The medium was replaced every 2–3 days. Upon reaching 80-90% confluence, cells were washed with Dulbecco´s Phosphate Buffered Saline (DPBS, Gibco, US), detached with 0.25% Trypsin-ethylenediaminetetraacetic acid (EDTA, Pan Biotech, Germany), and seeded in new flasks or treatment wells.

For the experiments DMEM/Ham´s F12 (1:1) supplemented with 1% Insulin-Transferrin-Selenium (ITS, Gibco, US) and 1% P/S was used to eliminate interfering effects of FCS and is labelled as serum-free medium or control (CTRL) medium in this study.

### 2.2 High hydrostatic pressure treatment and preparation of conditioned medium

HHP-treatment was performed in cryotubes (1,8 mL Nunc, Thermo Fisher Scientific, US). 1 × 10^6^ HNSCC cells (HNSCC16, UTSCC14, HNSCC46, PE/CA/PJ-15) were suspended in serum-free medium (DMEM/F12 + 1% ITS + 1% P/S) and added to the cryotube. The cryotubes were centrifuged at 120 x *g* for 8 min, filled with serum-free medium, and closed without air bubbles. The cryotubes were sealed with Parafilm M Laboratory Film (Pechiney Plastic Packaging Inc., US) and placed into water-filled, air-bubble-free centrifuge tubes. The sealed centrifuge tubes containing the samples were then placed in the glycol-filled pressure chamber of the high hydrostatic pressure device (Dustec Hochdrucktechnik GmbH, Germany). Cells were treated for 10 min at 0, 200 or 300 MPa respectively, with the temperature maintained at 20°C. The pressurization phase consisted of an initial pressure increase at +25 bar/s, followed by a slower ramp at +10 bar/s until the target pressure was reached. The target pressure was maintained for 10 min, after which decompression occurred at −25 bar/s. After treatment, the cryotubes were centrifuged again, incubated for 1 hour (h) at 37°C, and then the CM was transferred to a new cryotube. The CM was frozen at -80°C until further use in experiments.

CM of the different cell lines is labelled CM_HNSCC16,_ CM_UTSCC14,_ CM_HNSCC46_ and CM_PE/CA/PJ-15_ in this study.

### 2.3 Cytokine screening using the dot blot assay

The Human Cytokine Array C3 (RayBiotech Inc., USA) dot blot assay was used as a qualitative method to analyze the secretion of 42 cytokines, including key pro-inflammatory cytokines, chemokines, and growth factors. The assay was performed on thawed CM collected from pressure treated HNSCC16 (200 MPa and 300 MPa) and untreated control cells (0 MPa). The assay was conducted following the manufacturer’s protocol. Labeled proteins were visualized via enhanced chemiluminescence, using detection buffer and X-ray film exposure. Cytokines appeared as dots of varying intensity and size. Dot blot assay was performed once for qualitative cytokine screening to identify relevant targets for subsequent quantitative analysis.

### 2.4 Cytokine quantification in conditioned medium

Cytokine levels (IL-1α, IL-1β, Monocyte Chemoattractant Protein-1 (MCP-1), Stromal cell-derived factor 1 (SDF-1), IL-6, IL-8) of CM of treated cells (200 and 300 MPa) and control cells (0 MPa) were determined using the LEGENDplex Human Custom Panel (BioLegend, US) according to the manufacturer`s instructions. Briefly, CM were incubated with capture beads over night at 4°C on an orbital shaker. Next, detection antibody was added, and beads were incubated for 1 h at room temperature. After washing the beads, samples were measured using a BD Canto flow cytometer (BD Biosciences, US). Cytokine concentration was calculated of triplicates based on a standard curve using the BioLegend LEGENDplex data analysis software (version 2023-02-15, BioLegend, US). Quantification of cytokines was repeated at least four times per sample in technical triplicates.

### 2.5 Quantification of cellular proliferation

Cell counts over time were analyzed as a measure of cell proliferation using CASY Cell Counter (OMNI Life Science GmbH & Co. KG, Germany). HNSCC16 or UTSCC14 cells respectively were seeded in duplicates in a 24-well cell culture plate in serum-free medium. Medium was changed after 5 h and CM or control medium respectively were applied. Cells were incubated for 96 h and counted every 24 h. Each experiment was repeated at least four times in technical duplicates.

### 2.6 Analysis of cell migration potential

Tumor cell migration of HNSCC16 and UTSCC14 cells was evaluated by the scratch assay using Ibidi culture-insert 2 well system (Ibidi, Germany). Inserts were placed in a 24-well plate and 4 x 10^4^ HNSCC16 or 5 x 10^4^ UTSCC14 cells respectively were seeded in 100 μL serum-free medium in each insert chamber. After allowing the cells to attach overnight, the culture insert was removed to create a cell-free gap, and the cells were covered with CM or control medium respectively. Migrating cells were documented immediately after creation of the gap (0 h) at 10x magnification via live-cell-imaging (Carl Zeiss AG, Germany) under stable pressure of 5% CO_2_ in air at 37°C. The cell migration was recorded at the whole area of the gap at 2 h time intervals for 16 h. Evaluation was performed by measuring the cell-free area with ImageJ software (version 1.54 f, National Institutes of Health, US). Data was collected from at least three independent experiments each performed with four to six technical replicates.

### 2.7 Characterization of the tumor cell invasion potential

Tumor cell invasion potential of HNSCC16 and UTSCC14 cells was evaluated using the Boyden chamber assay. For this purpose, 5 x 10^4^ cells were seeded in triplicates in 800 μL serum-free medium on top of gelatine-coated filter (pore size 8 μm; it4ip S.A., Belgium). The bottom chamber was filled with 290 μL of CM or control medium respectively. The cells were incubated for 4 or 24 h in the chambers at 37°C and 5% CO_2_. After incubation time the filters were removed, and non-migrated cells were wiped away. Filters were then placed upside down on a glass slide and cells were fixed with 4% paraformaldehyde (Otto Fischar GmbH & Co. KG, Germany) for 10 min. Afterwards, filters were washed in DPBS and stained with Hoechst reagent (0.5 µg/mL; Sigma-Aldrich, US) for 10 min and placed on glass slides. The invaded cells were counted using a fluorescence microscope (Leica Microsystems GmbH, Germany) at 10x magnification, evaluating ten fields of view per filter. Each experiment was repeated at least four times in technical triplicates.

### 2.8 Spheroid growth assay

Three-dimensional (3D) spheroid formation was assessed using HNSCC16 cells. Cells were seeded into 12-well plates and treated with CM_HNSCC16_ (0 MPa and 300 MPa) or control medium respectively for priming the cells for 48 h. Subsequently, cells were detached and 1 × 10^5^ were seeded in a custom-fabricated polydimethylsiloxane (PDMS) µ-well insert made from 1600 kPa-stiff silicone. For this, Sylgard*®* 184 (PDMS) (VWR, USA*)* was used and mixed in various base (A) to crosslinker (B) ratios (R) (R; A to B, (w/w)). Mixing ratios were calculated from existing calibrations and substrates were prepared as described previously (47). Cross-linking occurred at 50°C overnight. After curing, respective elasticities were measured with nanoindentation. Effective Young’s modulus (E_eff_) of 1600 kPa (R; 10 to 1 – cartilage) were validated and used up to a deviation of 10%. Sterilization of elastomer substrates was performed by washing once with 1 mL of 98% isopropanol (IPA) and 1 mL of 1x DPBS. Afterwards, substrates were dried completely under sterile conditions for at least 1 h before use. The insert provides 64 uniform microcavities, each 500 µm in diameter, allowing for reproducible spheroid formation and quantitative analysis of 3D cell growth. Either CM or control medium was added to each chamber. Spheroid growth was monitored over a period of five days. Images were acquired at 24 h, 48 h, and 120 h using a fluorescence microscope (Carl Zeiss AG, Germany) at 10x magnification. Spheroid area and diameter were quantified using ImageJ software (version 1.54f, National Institutes of Health, USA). A total number of 62 spheroids per condition was analyzed.

### 2.9 Statistical analysis

Statistical analysis was performed using GraphPad PRISM software, version 8.0.1 (GraphPad Software, US). A p-value <0.05 was considered statistically significant. After proving the assumption of normality (Shapiro-Wilk test), one-way ANOVA with Tukey´s multiple comparison *post hoc* test was performed. If the normality test failed, the Kruskal-Wallis test with Dunn´s multiple comparison test was performed.

## 3 Results

This study identified and quantified cytokines associated with HHP-treatment of tumor cells. Additionally, the cytokine effects on the biological behavior of HNSCC tumor cells were analyzed, focusing on cell proliferation, migration, and invasion.

### 3.1 HHP-induced cytokine secretion

After treatment with HHP (0, 200, and 300 MPa), the CM from the tumor cells was analyzed for 42 cytokines using an antibody membrane assay, with a focus on pro-inflammatory cytokines (**Supplementary Figure 1**). All groups showed signals for IL-6, IL-8, MCP-1, and SDF-1. Additionally, the samples treated with 200 and 300 MPa showed signals for IL-1α and IL-1β.

### 3.2 Quantification of secreted cytokines

For detailed quantification of the identified cytokines, concentrations of IL-1α, IL-1β, MCP-1, SDF-1, IL-6, and IL-8 were determined using multiplex analysis.

**Figure 2:**
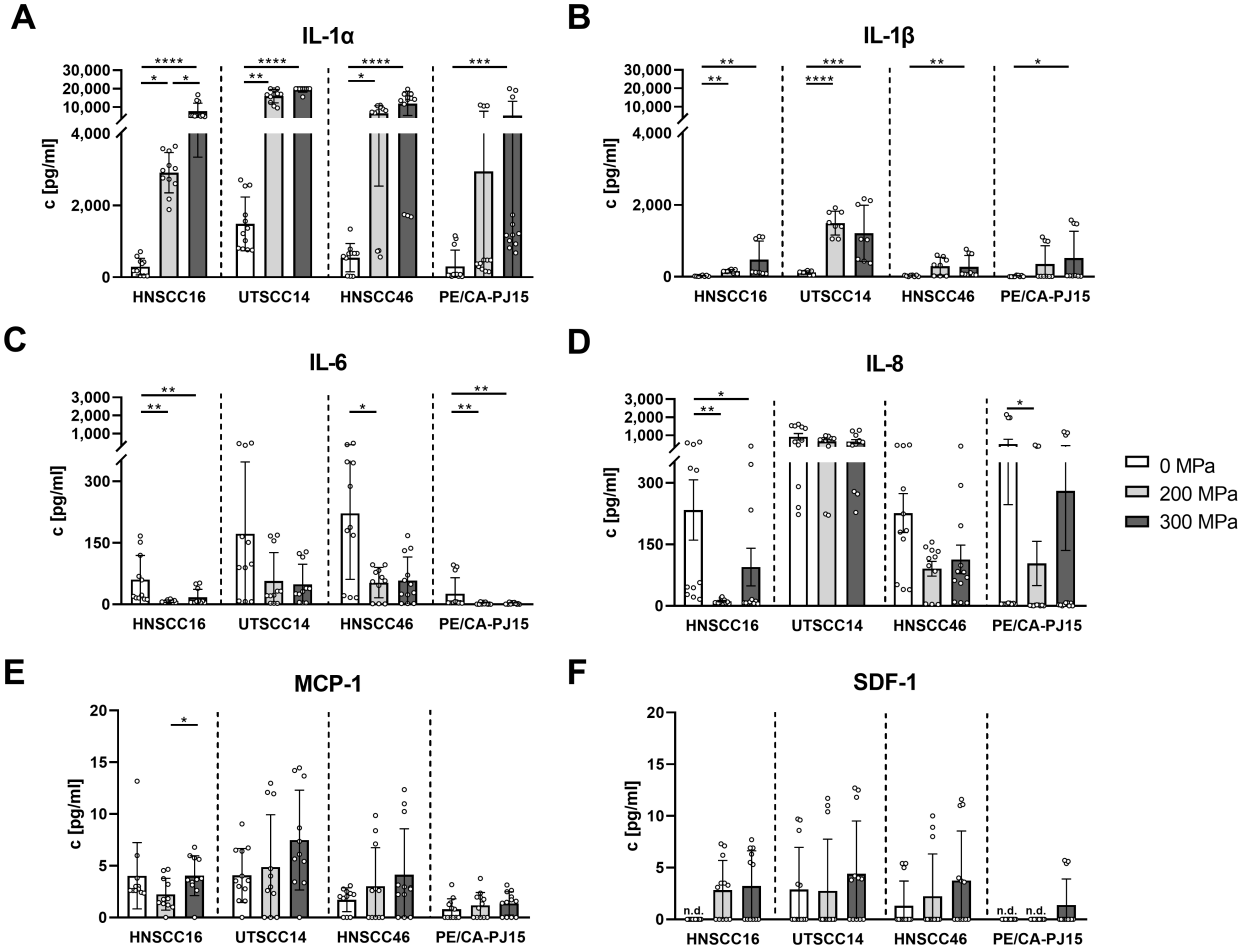
Cytokine quantification in conditioned medium (CM) of high hydrostatic pressure-treated tumor cells using multiplex analysis. HNSCC cells HNSCC16, UTSCC14, HNSCC46 and PE/CA/PJ15 were treated with 0, 200, 300 MPa. CM was analyzed in a multiplex assay for IL-1α **(A)**, IL-1β **(B)**, IL-6 **(C)**, IL-8 **(D)**, MCP-1 **(E)**, and SDF-1 **(F)**. Results are shown as means and standard deviations; n=8-12 (from four independent experiments). Statistical analysis was performed via one-way ANOVA and Tukey’s multiple comparison *post hoc* test; *p<0.05; **p<0.01, ***p<0.001; ****p<0.0001. n.d, not detected.

IL-1α (**Figure 2A**) levels increased significantly after HHP-treatment, particularly at 300 MPa, across all analyzed cell lines. Compared to the untreated control, IL-1α concentrations were elevated by 26-fold in CM_HNSCC16_ (p<0.0001), 13-fold in CM_UTSCC14_ (p<0.0001), 22-fold in CM_HNSCC46_ (p<0.0001), and 18-fold in CM_PE/CA/PJ-15_ (p=0.001). Treatment at 200 MPa also led to significant IL-1α increases in CM_HNSCC16_ (10-fold, p=0.0229), CM_UTSCC14_ (11-fold, p=0.0019), and CM_HNSCC46_ (12-fold, p=0.0254) compared to untreated controls. The concentration in the 300 MPa treated samples was higher than in the 200 MPa treated samples, with significant differences observed in CM_HNSCC16_ (2.7-fold increase, p=0.0229).

IL-1β (**Figure 2B**) followed a similar pattern, with significant increases at 300 MPa across all cell lines. Compared to controls, IL-1β concentrations increased by 24-fold in CM_HNSCC16_ (p=0.0021), 10-fold in CM_UTSCC14_ (p=0.0006), 11-fold in CM_HNSCC46_ (p=0.0042), and 34-fold in CM_PE/CA/PJ-15_ (p=0.0247). At 200 MPa, IL-1β levels were significantly elevated in CM_HNSCC16_ (7.5-fold, p=0.0021) and CM_UTSCC14_ (13-fold, p<0.0001) compared to untreated samples. Although IL-1β levels were higher at 300 MPa than 200 MPa in all cell lines, the differences were not statistically significant.

In contrast, IL-6 (**Figure 2C**) concentrations were lower in HHP-treated samples compared to controls across all cell lines. At 200 MPa, IL-6 levels decreased by 8-fold in CM_HNSCC16_ (p=0.0015), 4-fold in CM_HNSCC46_ (p=0.0211), and 14-fold in CM_PE/CA/PJ-15_ (p=0.0064). At 300 MPa, significant reductions were observed in CM_HNSCC16_ (3.6-fold, p=0.0042) and CM_PE/CA/PJ-15_ (14-fold, p=0.0100). No significant differences were detected between 200 MPa and 300 MPa treatments.

A similar trend was observed for IL-8 (**Figure 2D**), with significant decreases at 200 MPa in CM_HNSCC16_ (20-fold, p=0.0024) and CM_PE/CA/PJ-15_ (1.8-fold, p=0.0377) compared to controls. At 300 MPa, IL-8 levels were significantly lower than in untreated controls in CM_HNSCC16_ (2.5-fold reduction, p=0.0101).

MCP-1 (**Figure 2E**) showed a dose-dependent increase in CM_UTSCC14_, CM_HNSCC46_, and CM_PE/CA/PJ-15_. However, statistical significance was observed only between 200 MPa and 300 MPa in CM_HNSCC16_ (1.8-fold increase, p=0.0458).

SDF-1 (**Figure 2F**) levels remained low across all samples, showing a non-significant trend towards increased release with higher pressure in all cell lines.

### 3.3 Proliferation analysis of HNSCC cells after treatment with conditioned medium

The effect of cytokines released by HHP-treated HNSCC tumor cells on the proliferation of HNSCC16 and UTSCC14 cells was analyzed by cell counting.

**Figure 3:**
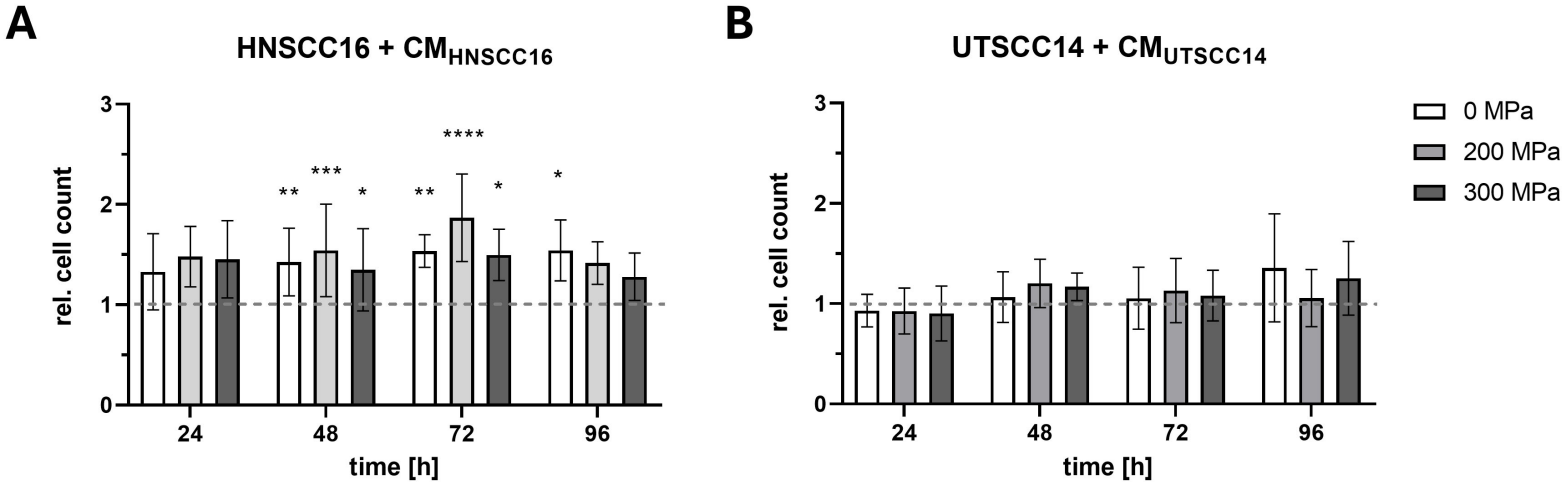
Proliferation analysis of HNSCC cells exposed to conditioned medium (CM). HNSCC16 **(A)** and UTSCC14 **(B)** cells were cultured for 96 h with CM (0, 200, and 300 MPa). Serum-free culture medium served as reference and was set to 1 and all experimental conditions were normalized to this control. Data are presented as means and standard deviations; n=5-8 (from four independent experiments). Statistical analysis: one-way ANOVA, Tukey’s multiple comparison *post hoc* test; *p<0.05; **p<0.01, ***p<0.001; ****p<0.0001.

HNSCC16 cell proliferation (**Figure 3A**) increased when cultured with CM_HNSCC16_, compared to the medium control. Cells cultured with 200 MPa-CM exhibited the highest proliferation rates, with significant increases of 1.54-fold after 48 h (p=0.0002) and 1.87-fold after 72 h (p<0.0001) compared to the medium control. Similarly, cells cultured with 300 MPa-CM showed significantly higher proliferation, with a 1.35-fold increase at 48 h (p=0.0368) and a 1.50-fold increase at 72 h (p=0.0261) relative to the medium control. 0 MPa-CM_HNSCC16_ treated cells also promoted cell growth, with significant proliferation increases of 1.43-fold at 48 h (p=0.0032), 1.54-fold at 72 h (p=0.0071), and 1.54-fold at 96 h (p=0.0320) compared to the control.

In contrast, no significant differences in the growth behavior of UTSCC14 cells were observed after culturing with their own CM (**Figure 3B**).

To evaluate the relevance of cytokine effects under more physiologically representative conditions, a 3D spheroid growth assay was performed using HNSCC16 cells (**Supplementary Figure 2**). Cells were preconditioned with 0 MPa-CM, 300 MPa-CM, or control medium for 48 h prior to seeding in custom-fabricated μ-well inserts. During five days of observation, spheroid diameter and area were monitored. While spheroid growth was slightly reduced in the 300 MPa-CM group compared to control and 0 MPa-CM, the overall differences were moderate, and no proliferative enhancement was observed, in contrast to the 2D proliferation data.

### 3.4 Migration analysis of HNSCC cells after treatment with conditioned medium

The effect of cytokines following HHP-treatment on HNSCC cell migration was evaluated using a scratch assay.

**Figure 4:**
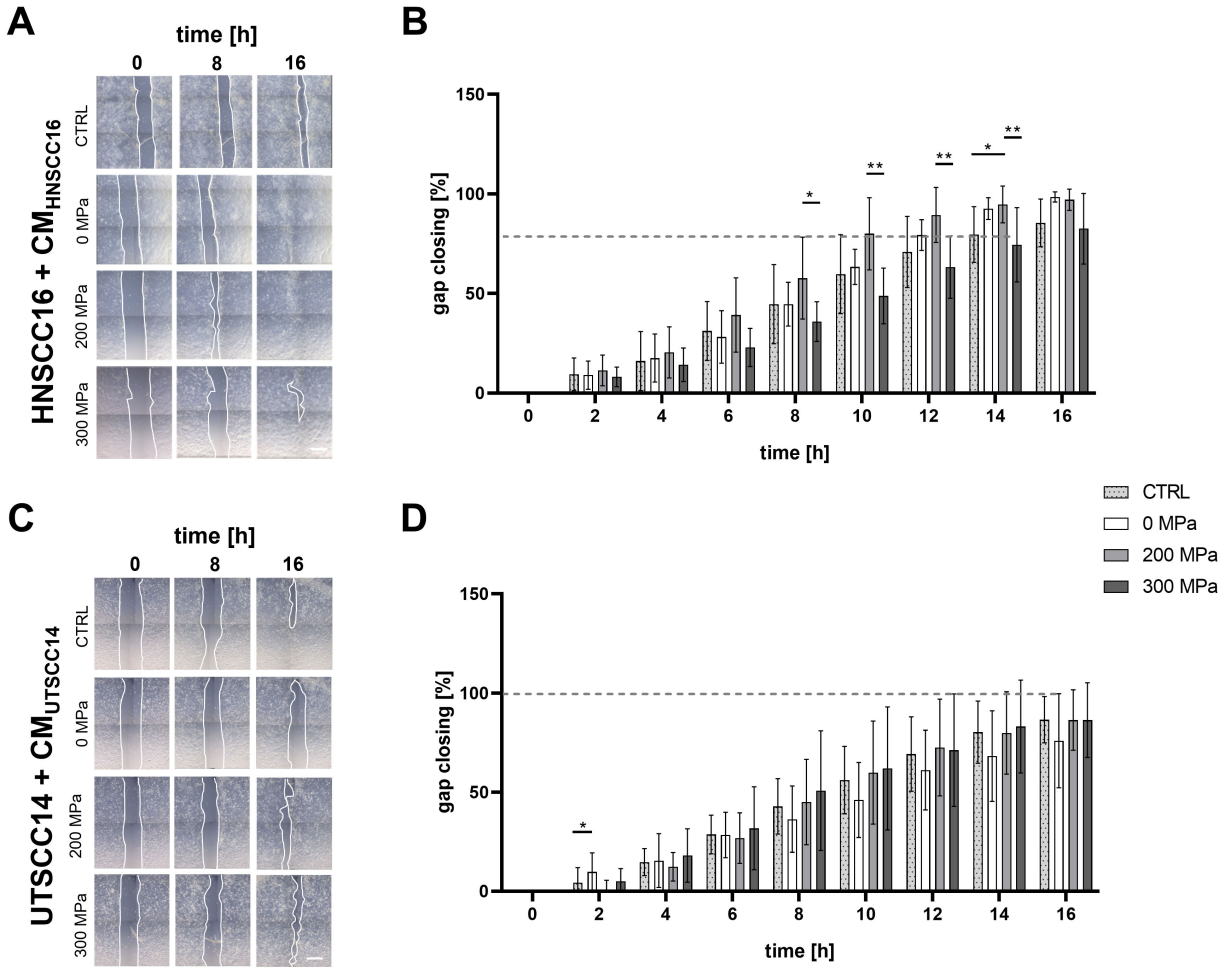
Migration analysis of HNSCC cells exposed to conditioned medium (CM). HNSCC16 and UTSCC14 cells were cultured for 48 h with their CM (0, 200, and 300 MPa). The conditioned cells were seeded into Ibidi wound healing inlays and allowed to adhere overnight. After inlays removal, the cells were cultured with the corresponding medium in an incubation chamber. Cell migration of HNSCC16 **(A)** and UTSCC14 **(C)** cells was documented via live-cell imaging. Migration speed was determined by measuring the area of the remaining gap, which was normalized to the area at time 0 h **(B, D)**. Serum-free culture medium served as reference (CTRL). Results are shown as means and standard deviations; n=10-11 (from three to four independent experiments). Statistical analysis: one-way ANOVA, Tukey’s multiple comparison *post hoc* test; *p<0.05; **p<0.01; scale bar: 500 µm.

The migration speed of HNSCC16 cells (**Figure 4A**) was highest when cultured with the 200 MPa-CM_HNSCC16_. After 16 h, the gap was nearly closed (97.08% ± 5.33%). The same was observed with the 0 MPa-CM (98.46% ± 2.56%). In contrast, the control and the 300 MPa-CM still showed an open gap after 16 h (85.45% ± 11.97% for CTRL; 82.52% ± 17.70% for 300 MPa) (**Figure 4B**). Significant differences between the 200 and 300 MPa conditions were observed after 8 h (57.71 ± 20.55% vs. 35.88 ± 9.95%; p=0.0119), 10 h (79.99 ± 18.08% vs. 48.79 ± 13.93%; p=0.0019), 12 h (89.42 ± 13.84% vs. 63.28 ± 15.64%; p=0.0094) and 14 h (94.70 ± 9.22% vs. 74.48 ± 18.69%; p=0.0047). After 14 h HNSCC16 cells treated with 200 MPa-CM showed significantly increased migration activity compared to cells treated with 0 MPa-CM (94.70 ± 9.22% vs. 79.64 ± 14.00%; p=0.0252)

Migration analysis of UTSCC14 cells (**Figure 4C**) following exposure to CM revealed no substantial differences between the various HHP-treatments. However, significant differences were observed between 0 MPa and 200 MPa treatments (9.75 ± 9.65% vs. 0.30 ± 5.26%; p=0.0277) within 2 h of migration analysis (**Figure 4D**). Between 8 and 16 h of incubation with CM, cells incubated with 0 MPa-CM exhibited reduced migratory activity compared to other conditions, although these differences were not statistically significant.

### 3.5 Invasion analysis of HNSCC cells after treatment with conditioned medium

The influence of cytokines in the CM after HHP-treatment on the invasion potential of HNSCC tumor cell lines was investigated using a Boyden chamber assay.

**Figure 5:**
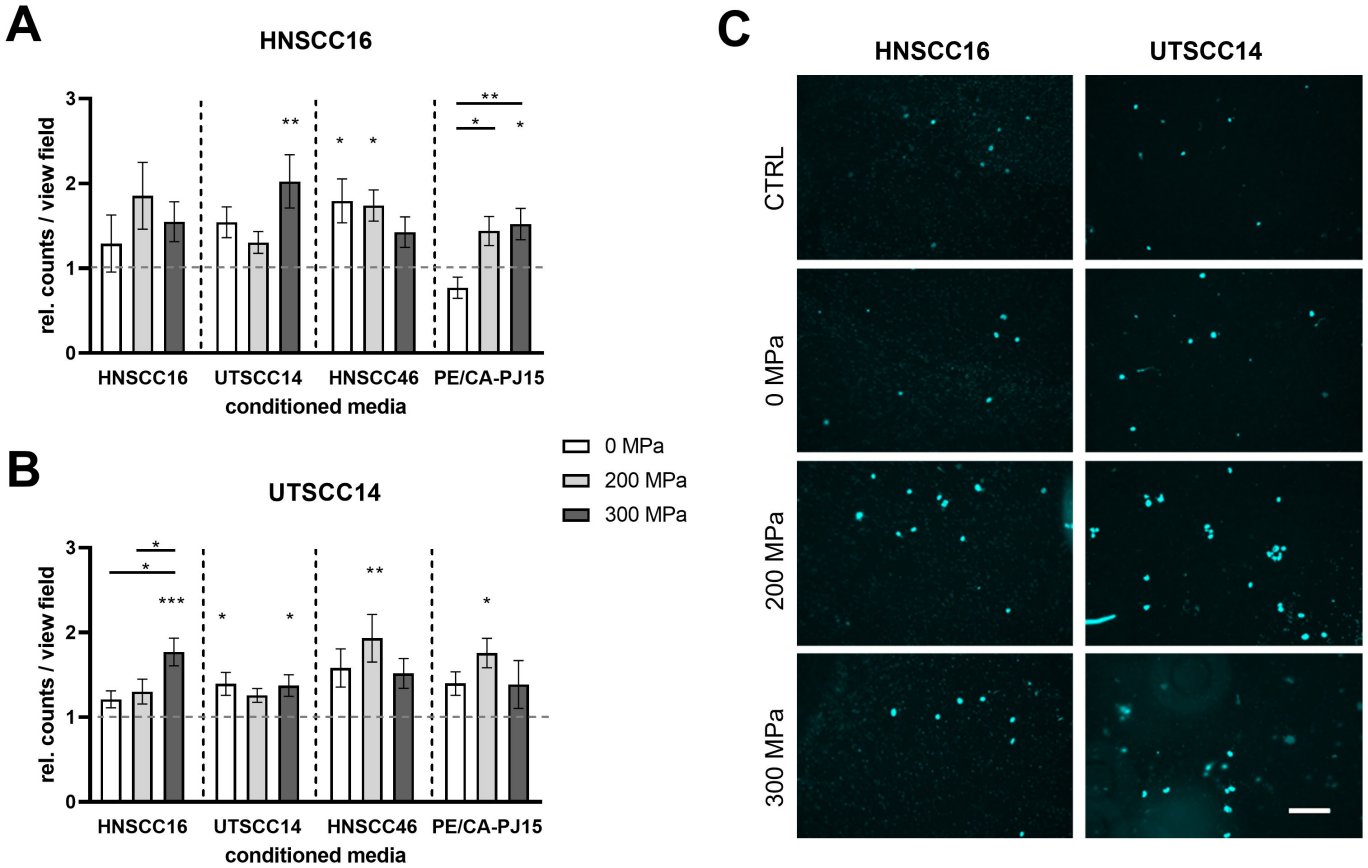
Invasion analysis of HNSCC cells exposed to conditioned medium (CM). HNSCC cell lines HNSCC16 and UTSCC14 were examined using a Boyden chamber with a pore size of 8 µm. The lower compartment contains the CM from their own or another HNSCC cell line after high hydrostatic pressure-treatment (0, 200 and 300 MPa). Serum-free culture medium served as reference and was set to 1. The number of HNSCC16 **(A)** and UTSCC14 **(B)** cells that migrated through the membrane was determined and normalized to the control. Representative pictures of migrated HNSCC16 and UTSCC14 cells through the membrane are shown **(C)**. Data are presented as means and standard deviations; n=8-12 (from four independent experiments). Statistical analysis: one-way ANOVA, Tukey’s multiple comparison *post hoc* test; *p<0.05; **p<0.01; ***p<0.001; scale bar: 100 µm.

HNSCC16 cells showed increased invasion when exposed to the CM compared to the medium control (**Figures 5A, C**). After stimulation with 300 MPa-CM_UTSCC14_, HNSCC16 cells demonstrated a 2.02-fold increase in invasion compared to the control (p=0.0036). Additionally, HNSCC16 cells exhibited significant increases in invasion with 0 MPa-CM_HNSCC46_ and 200 MPa-CM_HNSCC46_, showing 1.79-fold (p=0.0236) and 1.74-fold increases (p=0.0337), respectively. In the presence of CM_PE/CA/PJ-15_, HNSCC16 cells showed 1.52-fold increased invasion activity with 300 MPa compared to the control (p=0.0494), as well as 1.87-fold increases between 0 MPa and 200 MPa and 0 MPa and 300 MPa (p=0.0132 in both cases). Boyden chamber assay with a prolonged incubation time of 24 h also revealed a trend toward increased invasion in response to HHP-conditioned medium across all cell lines (**Supplementary Figure 3**) with a statistically significant increase observed for HNSCC16 cells treated with 0 MPa-CM_HNSCC46_ compared to the control (1.59-fold increase; p=0.0386).

Similarly, UTSCC14 cells demonstrated increased invasion potential when exposed to the CM, with all CM conditions (0, 200, and 300 MPa) showing higher invasion rates than the medium control (**Figures 5B, C**). When exposed to 300 MPa-CM_HNSCC16_, UTSCC14 cells showed a 1.77-fold increase in invasion activity compared to the control (p=0.0002). Significant differences were also observed between 0 MPa and 300 MPa (1.21-fold vs. 1.77-fold; p=0.0110) and 200 MPa and 300 MPa (1.30-fold vs. 1.77-fold; p=0.0498). Treatment with CM_UTSCC14_ resulted in a 1.39-fold increase for 0 MPa (p=0.0332) and 1.37-fold increase for 300 MPa (p=0.0462) compared to the control. Additionally, exposure to CM_HNSCC46_ at 200 MPa resulted in a 1.93-fold increase compared to the control (p=0.078). CM_PE/CA/PJ-15_ (200 MPa) led to a 1.76-fold increase in invasion compared to the control (p=0.0239). No significant differences in invasion activity were found between the 200 MPa and 300 MPa treatments for CM_PE/CA/PJ-15_.

## 4 Discussion

### 4.1 Oncological safety of HHP-treatment

Oncological safety is paramount in both autologous reconstruction of cartilage-infiltrating tumors and the development of whole-cell vaccines. This includes addressing the potential retention of cytokines and tumor antigens after HHP-treatment, which may influence surrounding cells, including residual tumor cells at the resection margin. In the context of whole-cell vaccines, certain cytokines may enhance immune responses, improving therapeutic outcomes. By identifying and characterizing cytokines and analyzing their effects on tumor cell proliferation, migration, and invasion, we aimed to evaluate the safety of HHP-treatment for clinical applications.

A critical factor in ensuring oncological safety is the choice of pressure levels used for devitalization. Previous findings demonstrated that 315 MPa is the threshold for effective devitalization of HNSCC cells (28). In unpublished data, this threshold was refined to 300 MPa, the pressure used in this study. Notably, when a pressure of 200 MPa is used, complete devitalization of tumor cells is not achieved. In this study, 200 MPa was also investigated to compare its effects on cytokine release and the associated biological responses with those observed at 300 MPa.

### 4.2 Structural integrity and autologous reimplantation

Cartilage subjected to HHP retains its structural integrity and can be revitalized with stem cells, maintaining both biomechanical properties and immunological compatibility (6, 48). These properties form the basis for the safe use of HHP-treated cartilage in reconstructive procedures after tumor resection. The ability to preserve cartilage integrity is crucial for autologous reimplantation, ensuring that the tissue retains its original function without compromising mechanical stability.

Compared to other tissue regeneration methods, HHP offers the advantage of maintaining extracellular matrix structures without introducing foreign materials or relying on decellularization processes. Other approaches, such as scaffold-based cartilage regeneration or synthetic implants, often lack the biomechanical fidelity required for long-term integration. HHP-treated cartilage, in contrast, allows for seamless incorporation into the surgical site, supporting functional restoration while eliminating the risk of reintroducing viable tumor cells.

### 4.3 Cytokine profiles and impact on tumor cells

Screening for 42 cytokines was performed using a dot blot assay, focusing primarily on pro-inflammatory cytokines with potential immunomodulatory effects. The dot blot identified IL-1α, IL-1β, MCP-1, SDF-1, IL-6, and IL-8 as key cytokines released from HHP-treated HNSCC cells, primarily pro-inflammatory in nature. IL-1α and IL-1β were found to promote epithelial-to-mesenchymal transition (EMT), invasion, and an immunosuppressive microenvironment through recruitment of Myeloid-derived suppressor cells (MDSCs) and tumor associated macrophages (TAMs), while IL-6 and IL-8 were associated with chronic inflammation, cellular proliferation, and tumor progression (49–51). Both IL-1 and IL-6 play pivotal roles in creating a tumor-promoting microenvironment, with chronic inflammation fostering DNA damage, cellular proliferation, and immune suppression, all of which contribute to cancer progression (52–54). The multiplex analyses demonstrated a dose-dependent release of IL-1α and IL-1β with increasing pressure, likely due to intracellular storage and membrane disruption caused by HHP. It was shown that upon proapoptotic signals, IL-1α migrates to the nucleus, binds chromatin, and remains non-inflammatory (55). In contrast, necrotic signals cause IL-1α to move to the cytosol, where its release acts as a damage-associated molecular pattern (DAMP), triggering inflammatory responses via IL-1 receptor type I (IL-1R1) (56). IL-6 and IL-8 concentrations were higher in untreated samples, likely due to continuous cytokine secretion by intact cells. HNSCC cells exhibit basal IL-6 expression driven by the constitutive activation of the NF-κB and STAT3 signaling pathways, while basal IL-8 expression is regulated primarily through the NF-κB pathway (57–59). Under stress conditions, such as inflammation or, in this study, hypoxia resulting from incubation in sealed cryo vials during and after HHP-treatment, as well as DNA damage induced by pressure treatment, a significant increase in IL-6 and IL-8 production was observed (60–62). SDF-1 and MCP-1, which are also associated with migration, invasion and tumor progression, showed comparatively very low concentrations in the multiplex analysis (63–65).

Functional assays revealed distinct biological effects of the cytokines released during HHP-treatment on other HNSCC cells. In scratch assays with HNSCC16 cells, CM from 300 MPa-treated cells slowed migration compared to 200 MPa-treated CM, consistent with the need for complete devitalization to minimize residual tumor vitality (28). In Boyden chamber assays, increased invasion potential was observed in both cell lines exposed to CM, regardless of the pressure applied. These observations were consistent after 4 h and 24 h incubation times in the Boyden chamber. However, no direct correlation between HHP amplitude and invasion rates could be established. HNSCC16 cells exposed to CM exhibited significantly increased proliferation, whereas UTSCC14 cells were not affected. This differential response likely reflects both the distinct biological origins and the genetic backgrounds of the two cell lines. UTSCC14, a long-established tongue carcinoma cell line, likely shows reduced microenvironmental sensitivity due to prolonged *in vitro* passaging (66). In contrast, PDX-derived cell lines, such as HNSCC16, preserve tumor heterogeneity and cytokine responsiveness (67, 68). Beyond origin, genetic differences further contribute to the observed functional divergence. While both cell lines harbor mutations in the *TP53* gene, UTSCC14 additionally carries a homozygous loss-of-function mutation in *CDKN2A*, whereas this gene is wild type in HNSCC16 (46, 69). The loss of *CDKN2A* disrupts the G1/S checkpoint, resulting in constitutive cell cycle progression and reduced sensitivity to external cues such as proinflammatory cytokines (70). Interestingly, while the proliferative effects of HHP-conditioned medium were clearly detectable in 2D monolayer cultures, these effects were not observed within the timeframe of our 3D spheroid assay. Spheroid growth of HNSCC16 cells pretreated with 300 MPa-CM remained comparable to the control group. This observation highlights the distinct characteristics and limitations of both systems. 2D cultures provide uniform exposure to soluble factors and allow sensitive detection of early, cytokine-driven effects on proliferation, making them particularly well suited for assessing short-term tumor cell activation and oncological safety. In contrast, 3D models more closely reflect *in vivo*-like tissue architecture, including diffusion barriers, gradients of oxygen and nutrients, and cell–matrix interactions, which may delay or attenuate cytokine effects. It is therefore possible that longer observation periods or additional 3D assay formats may be required to fully capture functional changes in such settings. Nevertheless, the absence of proliferative stimulation in the 3D model, even under cytokine-rich conditions, further supports the interpretation that HHP treatment at 300 MPa does not promote tumor cell regrowth. Together, these complementary findings strengthen the conclusion that HHP-treated tissues meet key oncological safety criteria *in vitro*.

### 4.4 Pro- and anti-tumorigenic effects

This study is the first to examine the cytokine profile in HHP-devitalized HNSCC. Our findings suggest that cytokines retained within devitalized tumor cells may interact with residual tumor cells at the resection margin. On the one hand, these cytokines could contribute to a pro-tumorigenic microenvironment by promoting tumor regrowth through mechanisms such as enhanced cell proliferation, angiogenesis, or EMT (71). On the other hand, it is conceivable that pro-inflammatory cytokines might attract immune effector cells, including cytotoxic T cells and natural killer (NK) cells, potentially supporting immune surveillance and aiding in the destruction of residual tumor cells (72, 73). However, this interpretation remains speculative in the absence of direct functional evidence. To clarify potential effects on immune cell recruitment, activation, or modulation, future investigations should incorporate functional assays, such as co-culture systems or *in vivo* models. The overall effect of the cytokines likely depends on multiple factors, including the specific cytokine composition, the viability of remaining tumor cells, and the immune status of the patient (74–76). Furthermore, the potential clinical impact of cytokines released from HHP-treated tumor cells must be considered in the context of their biological degradation kinetics and the procedural handling of devitalized tissue. Given the short half-life of cytokines (intracellular half-life for IL-1α: 15 h; IL-1β: 2.5 h (77), half-life in serum for IL-6: 15 h (78)), and additional washing steps during the preparation and implantation of devitalized grafts are likely to further reduce cytokine concentrations. Moreover, since the devitalized tissue is no longer metabolically active, no further cytokine production occurs post-implantation. Therefore, any paracrine influence of residual cytokines on surrounding viable tumor cells in the clinical setting is expected to be minimal and transient.

### 4.5 Clinical applications and perspectives

Standard treatments for squamous cell carcinoma include partial or complete organ removal, which significantly impacts function and aesthetics. When surgery is not feasible, radiotherapy is used, though it carries its own side effects. Combining surgery with radiotherapy is also an option. In the future, systemic therapies that target cancer cells throughout the body may be incorporated. Our approach to organ-preserving surgery with autologous cartilage reuse aims to minimize functional and aesthetic limitations by using HHP-treated autologous cartilage for reconstruction. The use of HHP-treated cartilage eliminates the risk of reintroducing viable tumor cells while maintaining safety for the patient.

Another possible field of application for tumor cells devitalized by HHP is whole cell vaccination, as it has already been shown that devitalization by HHP triggers immunogenic cell death (8, 35). Tumor cells devitalized by HHP can be used to generate immunogenic vaccines that elicit anti-tumor responses while maintaining a structurally intact antigenic profile (28, 30). In this context, cytokines released from HHP-treated tumor cells may positively influence the immune response, serving as adjuvants to enhance vaccine efficacy (79, 80). Pro-inflammatory cytokines play a crucial role as adjuvants in tumor vaccination strategies, enhancing the immune response against cancer cells. These cytokines improve the anti-tumor immune response enhancing antigen presentation, stimulating T cell responses or modulating the tumor microenvironment. In mouse models of subcutaneous melanoma and Lewis lung carcinoma, an IL-1β-based targeted cytokine was shown to safely enhance antitumor CD8+ T cell responses and synergize with other immunotherapies (81). Additionally, IL-1α and IL-1β have shown efficacy as mucosal adjuvants, promoting strong systemic and mucosal immune responses, which could be crucial for vaccines targeting pathogens like Human immunodeficiency virus (HIV) that infect mucosal surfaces (82). This approach aligns with current immunotherapeutic strategies, potentially complementing checkpoint inhibitors and adoptive T-cell therapies.

### 4.6 Limitations

This study investigated the oncological safety of HHP-treatment with regard to the autologous reimplantation of the treated tissue. Our findings offer a comprehensive overview of relevant, particularly pro-inflammatory, cytokines associated with HHP-treatment and their influence on other tumor cells. However, the potential influence of surrounding stem cells and fibroblasts that may transdifferentiate into cancer-associated fibroblasts (CAFs), supporting tumor progression, remains unclear and requires further exploration to fully confirm oncological safety (83, 84).

The use of CM following HHP-treatment allowed detailed cytokine profile analysis, identifying key factors involved in tumor progression. While our study provides a robust characterization using both commercially available and PDX-derived cell lines, these models may not fully recapitulate the tumor microenvironment or patient-specific heterogeneity. Further research involving primary tumor cells and proteome analysis could uncover additional mediators relevant to reimplantation and tumor vaccination. Expanding the cytokine screening approach could help identify previously unknown factors influencing clinical applications. Our findings are limited to *in vitro* models, which do not account for the complexity of the *in vivo* tumor microenvironment, immune interactions, and systemic factors. As an initial step toward more physiologically relevant modeling, we included a 3D spheroid assay using HNSCC16 cells. Further refinement and expansion of 3D models, including invasion assays and tumor-stroma co-culture systems, will be essential to fully assess the translational relevance of our findings and to confirm the oncological safety of HHP-treated materials under tissue-like conditions. To complement these efforts, future studies will incorporate *in vivo* models, including xenograft HNSCC mouse models, to investigate the oncological safety and immunological consequences of pressure-treated tumor material in a systemic context. These experiments will be crucial to determine whether the observed cytokine alterations influence tumor progression, or immune cell recruitment, thereby providing critical validation for the clinical use of HHP-treated tissue in oncological reconstructive surgery.

The oncological safety of HHP-treatment was the primary focus in this study, but its immunogenicity, crucial for whole-cell vaccine development, requires further study. While HHP-induced immunogenic cell death in HNSCC cells is established, the role of cytokines in immune activation remains unclear, necessitating follow-up research.

## 5 Conclusion

In summary, cytokines present in the conditioned medium were found to enhance the proliferation, migration, and invasion of HNSCC cells. However, our findings demonstrate that cytokines released during the HHP-treatment process, under a pressure of 300 MPa, do not promote tumor regrowth, as evidenced by functional assays. This underscores the oncological safety of HHP-treatment *in vitro*. The rapid degradation of these cytokines, along with their likely removal from tissues during post-treatment washing steps, suggests a favorable safety profile for HHP-treated cartilage in autologous reimplantation. However, further investigations are needed to validate these findings in clinical settings. Interestingly, the cytokines released during HHP-treatment may hold potential as adjuvants in whole-cell vaccines, contributing to enhanced immune responses. To fully understand the clinical implications of these findings and to explore the therapeutic potential of HHP-treated materials in oncology, further *in vitro* and *in vivo* studies are essential.

## Data Availability

The raw data supporting the conclusions of this article will be made available by the authors, without undue reservation.

## References

[1] Rivalain N Roquain J Demazeau G. Development of high hydrostatic pressure in biosciences: pressure effect on biological structures and potential applications in biotechnologies. Biotechnol Adv. (2010) 28:659–72. doi: 10.1016/j.biotechadv.2010.04.001, PMID: 20398747

[2] Waletzko-Hellwig J Pohl C Riese J Schlosser M Dau M Engel N. Effect of high hydrostatic pressure on human trabecular bone regarding cell death and matrix integrity. Front Bioeng Biotechnol. (2021) 9:730266. doi: 10.3389/fbioe.2021.730266, PMID: 34458245 PMC8387795

[3] Weiss EM Frey B Rödel F Herrmann M Schlücker E Voll RE. Ex vivo- and *in vivo*-induced dead tumor cells as modulators of antitumor responses. Ann N Y Acad Sci. (2010) 1209:109–17. doi: 10.1111/j.1749-6632.2010.05743.x, PMID: 20958323

[4] Frey B Janko C Ebel N Meister S Schlücker E Meyer-Pittroff R. Cells under pressure - treatment of eukaryotic cells with high hydrostatic pressure, from physiologic aspects to pressure induced cell death. Curr Med Chem. (2008) 15:2329–36. doi: 10.2174/092986708785909166, PMID: 18855663

[5] van de Sande MAJ Bovée JVMG van Domselaar M van Wijk MJ Sanders I Kuijper E. Successful disinfection of femoral head bone graft using high hydrostatic pressure. Cell Tissue Bank. (2018) 19:333–40. doi: 10.1007/s10561-017-9678-6, PMID: 29264694 PMC6133176

[6] Waletzko J Dau M Seyfarth A Springer A Frank M Bader R. Devitalizing effect of high hydrostatic pressure on human cells—Influence on cell death in osteoblasts and chondrocytes. Int J Mol Sci. (2020) 21:3836. doi: 10.3390/ijms21113836, PMID: 32481635 PMC7312382

[7] Diehl P Schmitt M Schauwecker J Eichelberg K Gollwitzer H Gradinger R. Effect of high hydrostatic pressure on biological properties of extracellular bone matrix proteins. Int J Mol Med. (2005) 16:285–9. doi: 10.3892/ijmm.16.2.285, PMID: 16012763

[8] Seitz C Rückert M Deloch L Weiss EM Utz S Izydor M. Tumor cell-based vaccine generated with high hydrostatic pressure synergizes with radiotherapy by generating a favorable anti-tumor immune microenvironment. Front Oncol. (2019) 9:805. doi: 10.3389/fonc.2019.00805, PMID: 31555582 PMC6722191

[9] Rendueles E Omer MK Alvseike O Alonso-Calleja C Capita R Prieto M. Microbiological food safety assessment of high hydrostatic pressure processing: A review. LWT - Food Sci Technol. (2011) 44:1251–60. doi: 10.1016/j.lwt.2010.11.001

[10] San Martín MF Barbosa-Cánovas GV Swanson BG. Food processing by high hydrostatic pressure. Crit Rev Food Sci Nutr. (2002) 42:627–45. doi: 10.1080/20024091054274, PMID: 12487422

[11] Yamamoto K. Food processing by high hydrostatic pressure. Biosci Biotechnol Biochem. (2017) 81:672–9. doi: 10.1080/09168451.2017.1281723, PMID: 28300504

[12] Diehl P Schauwecker J Mittelmeier W Schmitt M. High hydrostatic pressure, a novel approach in orthopedic surgical oncology to disinfect bone, tendons and cartilage. Anticancer Res. (2008) 28:3877–83., PMID: 19192644

[13] Kalle F Stadler VP Brach JK Grote VF Pohl C Schulz K. High hydrostatic pressure treatment for advanced tissue grafts in reconstructive head and neck surgery. J BioMed Mater Res A. (2024) 18(1):e37791. doi: 10.1002/jbm.a.37791, PMID: 39295278

[14] Firat C Gurlek A Aydin NE. Viability of cartilage grafts in various forms. J Craniofac Surg. (2011) 22:1666–70. doi: 10.1097/SCS.0b013e31822f3b1f, PMID: 21959409

[15] Liu Y Zhou G Cao Y. Recent progress in cartilage tissue engineering—Our experience and future directions. Engineering. (2017) 3:28–35. doi: 10.1016/J.ENG.2017.01.010

[16] Tarhan E Cakmak O Ozdemir BH Akdogan V Suren D. Comparison of AlloDerm, fat, fascia, cartilage, and dermal grafts in rabbits. Arch Facial Plast Surg. (2008) 10:187–93. doi: 10.1001/archfaci.10.3.187, PMID: 18490546

[17] Albrecht T Wallner F. Options for reconstruction after injuries in the head and neck region. HNO. (2023) 71:57–62. doi: 10.1007/s00106-022-01230-5, PMID: 36260106

[18] Strüder D Ebert J Kalle F Schraven SP Eichhorst L Mlynski R. Head and neck cancer: A study on the complex relationship between qoL and swallowing function. Curr Oncol Tor Ont. (2023) 30:10336–50. doi: 10.3390/curroncol30120753, PMID: 38132387 PMC10742452

[19] American Society of Clinical Oncology DG P SA L GS W WM M DJ A. American Society of Clinical Oncology clinical practice guideline for the use of larynx-preservation strategies in the treatment of laryngeal cancer. J Clin Oncol Off J Am Soc Clin Oncol. (2006) 24:3693–704. doi: 10.1200/JCO.2006.07.4559, PMID: 16832122

[20] Wagner MM Curé JK Caudell JJ Spencer SA Nabell LM Carroll WR. Prognostic significance of thyroid or cricoid cartilage invasion in laryngeal or hypopharyngeal cancer treated with organ preserving strategies. Radiat Oncol Lond Engl. (2012) 7:219. doi: 10.1186/1748-717X-7-219, PMID: 23256610 PMC3551796

[21] Patel UA Howell LK. Local response to chemoradiation in T4 larynx cancer with cartilage invasion. Laryngoscope. (2011) 121:106–10. doi: 10.1002/lary.21181, PMID: 21120838

[22] Terrell JE Ronis DL Fowler KE Bradford CR Chepeha DB Prince ME. Clinical predictors of quality of life in patients with head and neck cancer. Arch Otolaryngol Head Neck Surg. (2004) 130:401–8. doi: 10.1001/archotol.130.4.401, PMID: 15096421

[23] Jose J Moor JW Coatesworth AP Johnston C MacLennan K. Soft tissue deposits in neck dissections of patients with head and neck squamous cell carcinoma: prospective analysis of prevalence, survival, and its implications. Arch Otolaryngol Neck Surg. (2004) 130:157–60. doi: 10.1001/archotol.130.2.157, PMID: 14967743

[24] Barsouk A Aluru JS Rawla P Saginala K Barsouk A. Epidemiology, risk factors, and prevention of head and neck squamous cell carcinoma. Med Sci. (2023) 11:42. doi: 10.3390/medsci11020042, PMID: 37367741 PMC10304137

[25] Loos E Meulemans J Vranckx J Poorten VV Delaere P. Tracheal autotransplantation for functional reconstruction of extended hemilaryngectomy defects: A single-center experience in 30 patients. Ann Surg Oncol. (2016) 23:1674–83. doi: 10.1245/s10434-015-5033-y, PMID: 26714937

[26] Piazza C Paderno A Nicolai P. Conservative surgery for laryngeal chondrosarcoma: a review of the most recently proposed approaches. Curr Opin Otolaryngol Head Neck Surg. (2017) 25:93–100. doi: 10.1097/MOO.0000000000000337, PMID: 28059901

[27] Navach V Chu F Cattaneo A Zorzi S Scelsi D Ansarin M. Cartilage framework reconstruction after resection of thyroid cartilage chondrosarcoma: A case report. Otolaryngol Case Rep. (2017) 4:12–4. doi: 10.1016/j.xocr.2017.07.002

[28] Maletzki C Freiin Grote V Kalle F Kleitke T Zimpfer A Becker AS. Establishing safe high hydrostatic pressure devitalization thresholds for autologous head and neck cancer vaccination and reconstruction. Cell Death Discov. (2023) 9:1–11. doi: 10.1038/s41420-023-01671-z, PMID: 37872173 PMC10593744

[29] Adkins I Fucikova J Garg AD Agostinis P Špíšek R. Physical modalities inducing immunogenic tumor cell death for cancer immunotherapy. Oncoimmunology. (2014) 3:e968434. doi: 10.4161/21624011.2014.968434, PMID: 25964865 PMC4352954

[30] Urbanova L Hradilova N Moserova I Vosahlikova S Sadilkova L Hensler M. High hydrostatic pressure affects antigenic pool in tumor cells: Implication for dendritic cell-based cancer immunotherapy. Immunol Lett. (2017) 187:27–34. doi: 10.1016/j.imlet.2017.05.005, PMID: 28495513

[31] Yan S Liu K Mu L Liu J Tang W Liu B. Research and application of hydrostatic high pressure in tumor vaccines. Oncol Rep. (2021) 45:75. doi: 10.3892/or.2021.8026, PMID: 33760193 PMC8020208

[32] Mikyskova R Indrova M Stepanek I Kanchev I Bieblova J Vosahlikova S. Dendritic cells pulsed with tumor cells killed by high hydrostatic pressure inhibit prostate tumor growth in TRAMP mice. OncoImmunology. (2017) 6:e1362528. doi: 10.1080/2162402X.2017.1362528, PMID: 29209567 PMC5706615

[33] Mikyšková R Štěpánek I Indrová M Bieblová J Šímová J Truxová I. Dendritic cells pulsed with tumor cells killed by high hydrostatic pressure induce strong immune responses and display therapeutic effects both in murine TC-1 and TRAMP-C2 tumors when combined with docetaxel chemotherapy. Int J Oncol. (2016) 48:953–64. doi: 10.3892/ijo.2015.3314, PMID: 26718011 PMC4750542

[34] Hradilova N Sadilkova L Palata O Mysikova D Mrazkova H Lischke R. Generation of dendritic cell-based vaccine using high hydrostatic pressure for non-small cell lung cancer immunotherapy. PloS One. (2017) 12:e0171539. doi: 10.1371/journal.pone.0171539, PMID: 28187172 PMC5302789

[35] Fucikova J Moserova I Truxova I Hermanova I Vancurova I Partlova S. High hydrostatic pressure induces immunogenic cell death in human tumor cells. Int J Cancer. (2014) 135:1165–77. doi: 10.1002/ijc.28766, PMID: 24500981

[36] Eisenthal A Ramakrishna V Skornick Y Shinitzky M. Induction of cell-mediated immunity against B16-BL6 melanoma in mice vaccinated with cells modified by hydrostatic pressure and chemical crosslinking. Cancer Immunol Immunother CII. (1993) 36:300–6. doi: 10.1007/BF01741168, PMID: 8477416 PMC11041093

[37] Weiss EM Meister S Janko C Ebel N Schlücker E Meyer-Pittroff R. High hydrostatic pressure treatment generates inactivated mammalian tumor cells with immunogeneic features. J Immunotoxicol. (2010) 7:194–204. doi: 10.3109/15476911003657414, PMID: 20205624

[38] Rückert M Deloch L Frey B Schlücker E Fietkau R Gaipl US. Combinations of radiotherapy with vaccination and immune checkpoint inhibition differently affect primary and abscopal tumor growth and the tumor microenvironment. Cancers. (2021) 13:714. doi: 10.3390/cancers13040714, PMID: 33572437 PMC7916259

[39] Li K Zeng X Liu P Zeng X Lv J Qiu S. The role of inflammation-associated factors in head and neck squamous cell carcinoma. J Inflammation Res. (2023) 16:4301–18. doi: 10.2147/JIR.S428358, PMID: 37791117 PMC10544098

[40] Landskron G la FMD Thuwajit P Thuwajit C Hermoso MA. Chronic inflammation and cytokines in the tumor microenvironment. J Immunol Res. (2014) 2014:149185. doi: 10.1155/2014/149185, PMID: 24901008 PMC4036716

[41] Briukhovetska D Dörr J Endres S Libby P Dinarello CA Kobold S. Interleukins in cancer: from biology to therapy. Nat Rev Cancer. (2021) 21:481–99. doi: 10.1038/s41568-021-00363-z, PMID: 34083781 PMC8173513

[42] Zhao T Cai Y Jiang Y He X Wei Y Yu Y. Vaccine adjuvants: mechanisms and platforms. Signal Transduct Target Ther. (2023) 8:1–24. doi: 10.1038/s41392-023-01557-7, PMID: 37468460 PMC10356842

[43] Paston SJ Brentville VA Symonds P Durrant LG. Cancer vaccines, adjuvants, and delivery systems. Front Immunol. (2021) 12:627932. doi: 10.3389/fimmu.2021.627932, PMID: 33859638 PMC8042385

[44] Kurane S Arca MT Aruga A Krinock RA Krauss JC Chang AE. Cytokines as an adjuvant to tumor vaccines: efficacy of local methods of delivery. Ann Surg Oncol. (1997) 4:579–85. doi: 10.1007/BF02305540, PMID: 9367025

[45] Cuzzubbo S Mangsbo S Nagarajan D Habra K Pockley AG McArdle SEB. Cancer vaccines: adjuvant potency, importance of age, lifestyle, and treatments. Front Immunol. (2020) 11:615240. doi: 10.3389/fimmu.2020.615240, PMID: 33679703 PMC7927599

[46] Schoenwaelder N Krause M Freitag T Schneider B Zonnur S Zimpfer A. Preclinical head and neck squamous cell carcinoma models for combined targeted therapy approaches. Cancers. (2022) 14:2484. doi: 10.3390/cancers14102484, PMID: 35626088 PMC9139292

[47] Cesa CM Kirchgessner N Mayer D Schwarz US Hoffmann B Merkel R. Micropatterned silicone elastomer substrates for high resolution analysis of cellular force patterns. Rev Sci Instrum. (2007) 78:034301. doi: 10.1063/1.2712870, PMID: 17411201

[48] Hiemer B Genz B Jonitz-Heincke A Pasold J Wree A Dommerich S. Devitalisation of human cartilage by high hydrostatic pressure treatment: Subsequent cultivation of chondrocytes and mesenchymal stem cells on the devitalised tissue. Sci Rep. (2016) 6::33747. doi: 10.1038/srep33747, PMID: 27671122 PMC5037397

[49] Niklander SE Murdoch C Hunter KD. IL-1/IL-1R signaling in head and neck cancer. Front Oral Health. (2021) 2:722676. doi: 10.3389/froh.2021.722676, PMID: 35048046 PMC8757896

[50] Chew V Toh HC Abastado JP. Immune microenvironment in tumor progression: characteristics and challenges for therapy. J Oncol. (2012) 2012:608406. doi: 10.1155/2012/608406, PMID: 22927846 PMC3423944

[51] Guo B Fu S Zhang J Liu B Li Z. Targeting inflammasome/IL-1 pathways for cancer immunotherapy. Sci Rep. (2016) 6:36107. doi: 10.1038/srep36107, PMID: 27786298 PMC5082376

[52] Zhao H Wu L Yan G Chen Y Zhou M Wu Y. Inflammation and tumor progression: signaling pathways and targeted intervention. Signal Transduct Target Ther. (2021) 6:263. doi: 10.1038/s41392-021-00658-5, PMID: 34248142 PMC8273155

[53] Wen Y Zhu Y Zhang C Yang X Gao Y Li M. Chronic inflammation, cancer development and immunotherapy. Front Pharmacol. (2022) 13:1040163. doi: 10.3389/fphar.2022.1040163, PMID: 36313280 PMC9614255

[54] Kay J Thadhani E Samson L Engelward B. Inflammation-induced DNA damage, mutations and cancer. DNA Repair. (2019) 83:102673. doi: 10.1016/j.dnarep.2019.102673, PMID: 31387777 PMC6801086

[55] Cohen I Rider P Carmi Y Braiman A Dotan S White MR. Differential release of chromatin-bound IL-1α discriminates between necrotic and apoptotic cell death by the ability to induce sterile inflammation. Proc Natl Acad Sci. (2010) 107:2574–9. doi: 10.1073/pnas.0915018107, PMID: 20133797 PMC2823886

[56] Di Paolo NC Shayakhmetov DM. Interleukin 1α and the inflammatory process. Nat Immunol. (2016) 17:906–13. doi: 10.1038/ni.3503, PMID: 27434011 PMC5152572

[57] Squarize CH Castilho RM Sriuranpong V Pinto DS Gutkind JS. Molecular cross-talk between the NFκB and STAT3 signaling pathways in head and neck squamous cell carcinoma. Neoplasia N Y N. (2006) 8:733–46. doi: 10.1593/neo.06274, PMID: 16984731 PMC1584297

[58] Riebe C Pries R Schroeder KN Wollenberg B. Phosphorylation of STAT3 in Head and Neck Cancer Requires p38 MAPKinase, whereas Phosphorylation of STAT1 Occurs via a Different Signaling Pathway. Anticancer Res. (2011) 31(11):3819–25., PMID: 22110204

[59] Chan LP Liu C Chiang FY Wang LF Lee KW Chen WT. IL-8 promotes inflammatory mediators and stimulates activation of p38 MAPK/ERK-NF-&x03BA;B pathway and reduction of JNK in HNSCC. Oncotarget. (2017) 8:56375–88. doi: 10.18632/oncotarget.16914, PMID: 28915597 PMC5593568

[60] Cohen AN Veena MS Srivatsan ES Wang MB. Suppression of interleukin 6 and 8 production in head and neck cancer cells with curcumin via inhibition of Iκβ Kinase. Arch Otolaryngol Neck Surg. (2009) 135:190–7. doi: 10.1001/archotol.135.2.190, PMID: 19221248

[61] Xu Q Ma H Chang H Feng Z Zhang C Yang X. The interaction of interleukin-8 and PTEN inactivation promotes the Malignant progression of head and neck squamous cell carcinoma via the STAT3 pathway. Cell Death Dis. (2020) 11:1–14. doi: 10.1038/s41419-020-2627-5, PMID: 32471980 PMC7260373

[62] Nisar H Brauny M Labonté FM Schmitz C Konda B Hellweg CE. and inflammatory response of p53 null H358 non-small cell lung cancer cells to X-ray exposure under chronic hypoxia. Int J Mol Sci. (2024) 25:12590. doi: 10.3390/ijms252312590, PMID: 39684302 PMC11641747

[63] Yoshimura T Howard OMZ Ito T Kuwabara M Matsukawa A Chen K. Monocyte chemoattractant protein-1/CCL2 produced by stromal cells promotes lung metastasis of 4T1 murine breast cancer cells. PloS One. (2013) 8:e58791. doi: 10.1371/journal.pone.0058791, PMID: 23527025 PMC3601078

[64] Wang H Zhang Q Kong H Zeng Y Hao M Yu T. Monocyte chemotactic protein-1 expression as a prognosic biomarker in patients with solid tumor: a meta analysis. Int J Clin Exp Pathol. (2014) 7:3876–86., PMID: 25120764 PMC4128999

[65] Teng F Tian WY Wang YM Zhang YF Guo F Zhao J. Cancer-associated fibroblasts promote the progression of endometrial cancer via the SDF-1/CXCR4 axis. J Hematol OncolJ Hematol Oncol. (2016) 9:8. doi: 10.1186/s13045-015-0231-4, PMID: 26851944 PMC4744391

[66] Johansson N Airola K Grénman R Kariniemi AL Saarialho-Kere U Kähäri VM. Expression of collagenase-3 (matrix metalloproteinase-13) in squamous cell carcinomas of the head and neck. Am J Pathol. (1997) 151:499–508. 9250162 PMC1857999

[67] Jin J Yoshimura K Sewastjanow-Silva M Song S Ajani JA. Challenges and prospects of patient-derived xenografts for cancer research. Cancers. (2023) 15:4352. doi: 10.3390/cancers15174352, PMID: 37686627 PMC10486659

[68] Idrisova KF Simon HU Gomzikova MO. Role of patient-derived models of cancer in translational oncology. Cancers. (2022) 15:139. doi: 10.3390/cancers15010139, PMID: 36612135 PMC9817860

[69] Lepikhova T Karhemo PR Louhimo R Yadav B Murumägi A Kulesskiy E. Drug-sensitivity screening and genomic characterization of 45 HPV-negative head and neck carcinoma cell lines for novel biomarkers of drug efficacy. Mol Cancer Ther. (2018) 17:2060–71. doi: 10.1158/1535-7163.MCT-17-0733, PMID: 29970484

[70] Zhao R Choi BY Lee MH Bode AM Dong Z. Implications of genetic and epigenetic alterations of CDKN2A (p16(INK4a)) in cancer. EBioMedicine. (2016) 8:30–9. doi: 10.1016/j.ebiom.2016.04.017, PMID: 27428416 PMC4919535

[71] Ostrand-Rosenberg S. Immune surveillance: a balance between protumor and antitumor immunity. Curr Opin Genet Dev. (2008) 18:11–8. doi: 10.1016/j.gde.2007.12.007, PMID: 18308558 PMC2699403

[72] Galli F Aguilera JV Palermo B Markovic SN Nisticò P Signore A. Relevance of immune cell and tumor microenvironment imaging in the new era of immunotherapy. J Exp Clin Cancer Res. (2020) 39:89. doi: 10.1186/s13046-020-01586-y, PMID: 32423420 PMC7236372

[73] Tang T Huang X Zhang G Hong Z Bai X Liang T. Advantages of targeting the tumor immune microenvironment over blocking immune checkpoint in cancer immunotherapy. Signal Transduct Target Ther. (2021) 6:1–13. doi: 10.1038/s41392-020-00449-4, PMID: 33608497 PMC7896069

[74] Kartikasari AER Huertas CS Mitchell A Plebanski M. Tumor-induced inflammatory cytokines and the emerging diagnostic devices for cancer detection and prognosis. Front Oncol. (2021) 11:692142. doi: 10.3389/fonc.2021.692142, PMID: 34307156 PMC8294036

[75] Yi M Li T Niu M Zhang H Wu Y Wu K. Targeting cytokine and chemokine signaling pathways for cancer therapy. Signal Transduct Target Ther. (2024) 9:1–48. doi: 10.1038/s41392-024-01868-3, PMID: 39034318 PMC11275440

[76] Lin WW Karin M. A cytokine-mediated link between innate immunity, inflammation, and cancer. J Clin Invest. (2007) 117:1175–83. doi: 10.1172/JCI31537, PMID: 17476347 PMC1857251

[77] Hazuda DJ Lee JC Young PR. The kinetics of interleukin 1 secretion from activated monocytes. Differences between interleukin 1 alpha and interleukin 1 beta. . J Biol Chem. (1988) 263:8473–9. doi: 10.1016/S0021-9258(18)68502-3, PMID: 3259579

[78] Wirtz DC Heller KD Miltner O Zilkens KW Wolff JM. Interleukin-6: a potential inflammatory marker after total joint replacement. Int Orthop. (2000) 24:194–6. doi: 10.1007/s002640000136, PMID: 11081839 PMC3619890

[79] Wang E Long B Wang K Wang J He Y Wang X. Interleukin-8 holds promise to serve as a molecular adjuvant in DNA vaccination model against Streptococcus iniae infection in fish. Oncotarget. (2016) 7:83938–50. doi: 10.18632/oncotarget.13728, PMID: 27911873 PMC5356636

[80] Wang E Wang J Long B Wang K He Y Yang Q. Molecular cloning, expression and the adjuvant effects of interleukin-8 of channel catfish (Ictalurus Punctatus) against Streptococcus iniae. Sci Rep. (2016) 6:29310. doi: 10.1038/srep29310, PMID: 27373470 PMC4931690

[81] Van Den Eeckhout B Huyghe L Van Lint S Burg E Plaisance S Peelman F. Selective IL-1 activity on CD8+ T cells empowers antitumor immunity and synergizes with neovasculature-targeted TNF for full tumor eradication. J Immunother Cancer. (2021) 9:e003293. doi: 10.1136/jitc-2021-003293, PMID: 34772757 PMC8593706

[82] Staats HF Ennis FA Jr. IL-1 is an effective adjuvant for mucosal and systemic immune responses when coadministered with protein immunogens1. J Immunol. (1999) 162:6141–7. doi: 10.4049/jimmunol.162.10.6141, PMID: 10229857

[83] Wright K Ly T Kriet M Czirok A Thomas SM. Cancer-associated fibroblasts: master tumor microenvironment modifiers. Cancers. (2023) 15:1899. doi: 10.3390/cancers15061899, PMID: 36980785 PMC10047485

[84] Mao X Xu J Wang W Liang C Hua J Liu J. Crosstalk between cancer-associated fibroblasts and immune cells in the tumor microenvironment: new findings and future perspectives. Mol Cancer. (2021) 20:131. doi: 10.1186/s12943-021-01428-1, PMID: 34635121 PMC8504100

