# Supplementary material for: Impact of high hydrostatic pressure on the cytokine profile and head and neck cancer cell behavior: implications for oncological safety
Source: Front Immunol. 2025 Jul 28;16:1581014. doi: 10.3389/fimmu.2025.1581014 (PMC12336235; doi:10.3389/fimmu.2025.1581014)
Supplement: Supplementary file 1 [file DataSheet1.pdf]

Supplementary figures

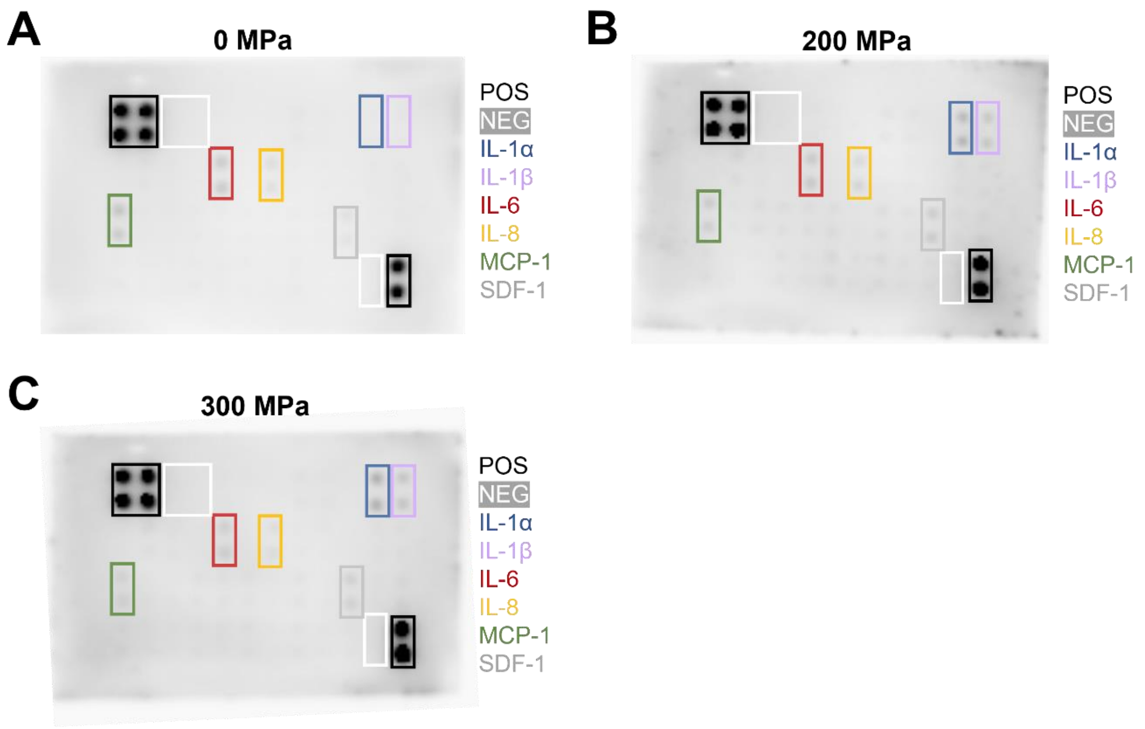

Supplementary Figure 1: Identification of relevant cytokines associated with high hydrostatic pressure-treated tumor cells. HNSCC16 cells were treated with 0 (**A**), 200 (**B**), 300 MPa (**C**). Conditioned medium was analyzed in a dot blot assay with correlation of the dot intensity to the respective cytokine concentration. Dot blot assay was performed once for qualitative cytokine screening to identify relevant targets for subsequent quantitative analysis.

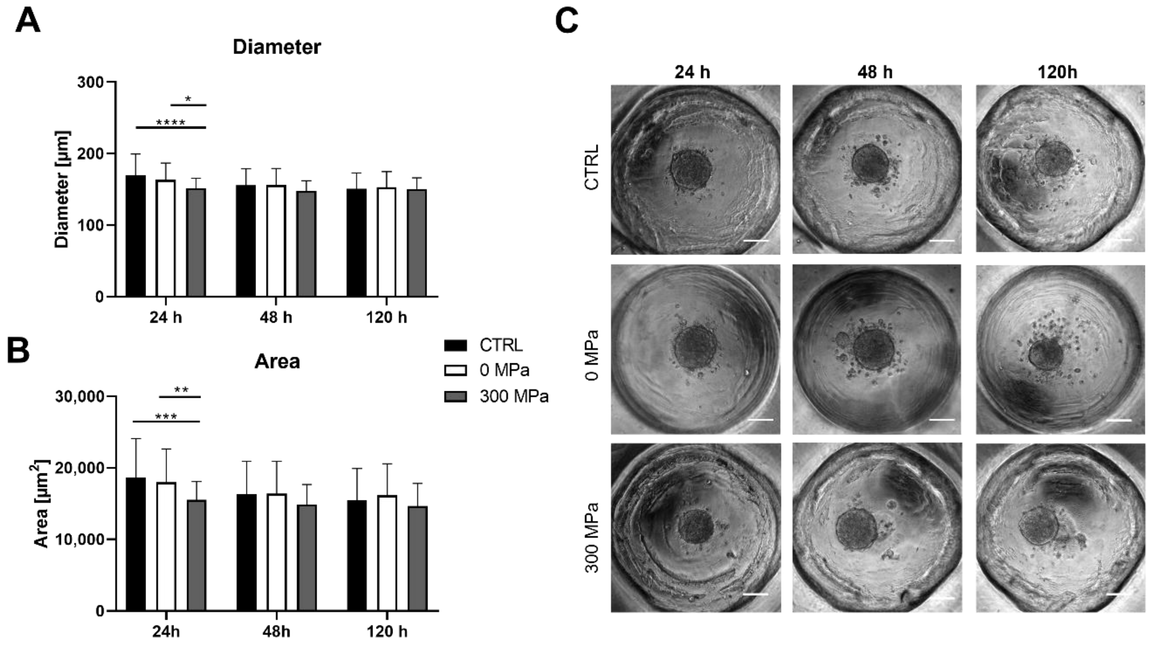

Supplementary Figure 2: Spheroid growth analysis of HNSCC16 cells exposed to conditioned medium (CM). HNSCC16 was cultured for 48 h in CM (0 and 300 MPa) and control medium, respectively. The conditioned cells were seeded into in house fabricated µ-well culture inserts. Spheroid growth was observed over five days and progress was documented for 24, 48 and 120 h. Spheroid growth was determined by measuring the diameter (**A**) and area (**B**) of each spheroid. Representative images of spheroids are shown (**C**). Results are shown as means

and standard deviations; n=62. Statistical analysis: one-way ANOVA, Tukey's multiple comparison post hoc test; *p<0.05; **p<0.01, ***p<0.001, ****p<0.0001; scale bar: 100 µm.

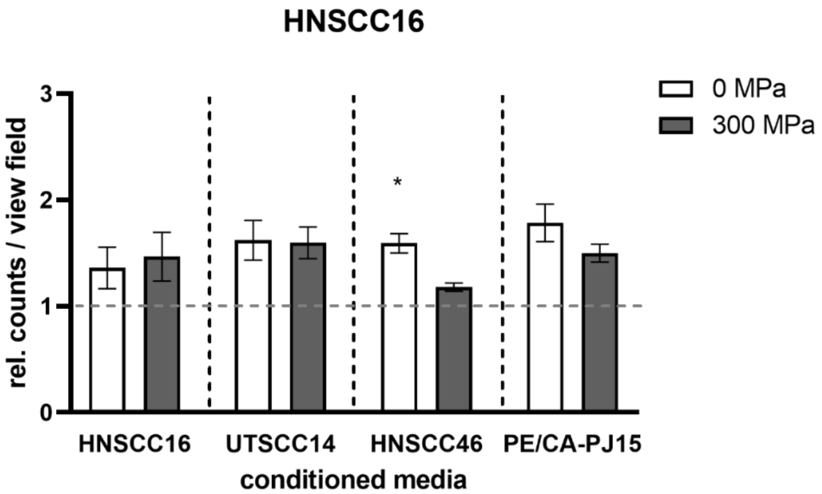

Supplementary Figure 3: Invasion rates of HNSCC16 cells exposed to conditioned medium (CM) for prolonged incubation time (24 h). The HNSCC cell line HNSCC16 was examined using a Boyden chamber with a pore size of 8 µm. The lower compartment contains the CM from their own or another HNSCC cell line after high hydrostatic pressure-treatment (0 and 300 MPa). Serum-free culture medium served as reference and was set to 1 (dashed line). The number of HNSCC16 cells that invaded through the membrane was determined and normalized to the serum-free medium control. Data are presented as means and standard deviations; n=5-6 (from three independent experiments). Statistical analysis: Kruskal-Wallis test with Dunn's multiple comparison test; *p<0.05.
